# Sensory-mediated “arts on prescription”: emotional arousal mechanisms through Portuguese Azulejo craft workshop

**DOI:** 10.3389/fpsyg.2025.1595856

**Published:** 2025-07-15

**Authors:** Jing Xu, Qianyao Wang, Aijia Zhang, Xuexing Luo, Guanghui Huang

**Affiliations:** ^1^Faculty of Humanities and Arts, Macau University of Science and Technology, Macau, China; ^2^Zhuhai M.U.S.T. Science and Technology Research Institute, Zhuhai, Guangdong, China

**Keywords:** arts therapy, multisensory, craft workshop, emotional arousal, Azulejo, behavioral healing mechanisms

## Abstract

**Background:**

This study investigates the therapeutic potential of the Portuguese Azulejo craft within an Arts on Prescription framework, focusing on how sensory engagement across creative stages supports the regulation of a positive emotional state, suggesting a potential U-shaped trajectory (with calming early phases and a spike of excitement in the final phase) in Portuguese Azulejo workshops. Furthermore, the study suggests a preliminary empirical relationship between MBTI personality types and color-emotion associations. Results demonstrate that multisensory engagement enhances positive affect, well-being, cultural cognition, and culture preservation intent, establishing a cognition-emotion-behaviour model for cross-cultural art interventions.

**Methods:**

Twenty-five participants (Mean age = 34.25) took part in a cross-cultural summer workshop. Emotional states were measured pre- and post-workshop using the Cultural Cognition and Culture Preservation intent, the Subjective Happiness Scale, and the PANAS. The Wilcoxon signed-rank test assessed changes in emotion (*p* < 0.05). Due to sample size limitations, Pearson correlations were used to examine associations between emotional shifts and related factors (cultural cognition, culture preservation intent, well-being and affect). Spearman rank correlations were conducted to assess the relationship between creative stages and emotional response, and also attempt to analyze and compare the Myers-Briggs Type Indicator (MBTI) with the colors and emotions used in the works to dissect the impact of personality traits on emotions in the craft experience.

**Results:**

Post-intervention, participants demonstrated significant improvements in cultural cognition (*p* < 0.001), subjective well-being (*p* = 0.003), and cultural preservation intent (*p* < 0.001). Positive affect, as measured by the PANAS, increased significantly (*p* < 0.001), while negative affect did not reach statistical significance (*p* = 0.16). Emotional responses varied across production stages, with initial tactile phases eliciting anticipation, mid-stage emotions stabilizing, and late-stage fluctuations linked to outcome expectations. Furthermore, participants were influenced by different patterns of MBTI personality types in terms of color use and emotional expression.

**Discussion:**

The study underscores the therapeutic value of multisensory craft experiences, particularly the role of tactile-visual synergy in emotional regulation. Cross-cultural craft workshops support cognitive engagement, emotional well-being, and protective behaviors, highlighting their potential as group-based therapeutic interventions.

## 1 Introduction

**Figure 1:**
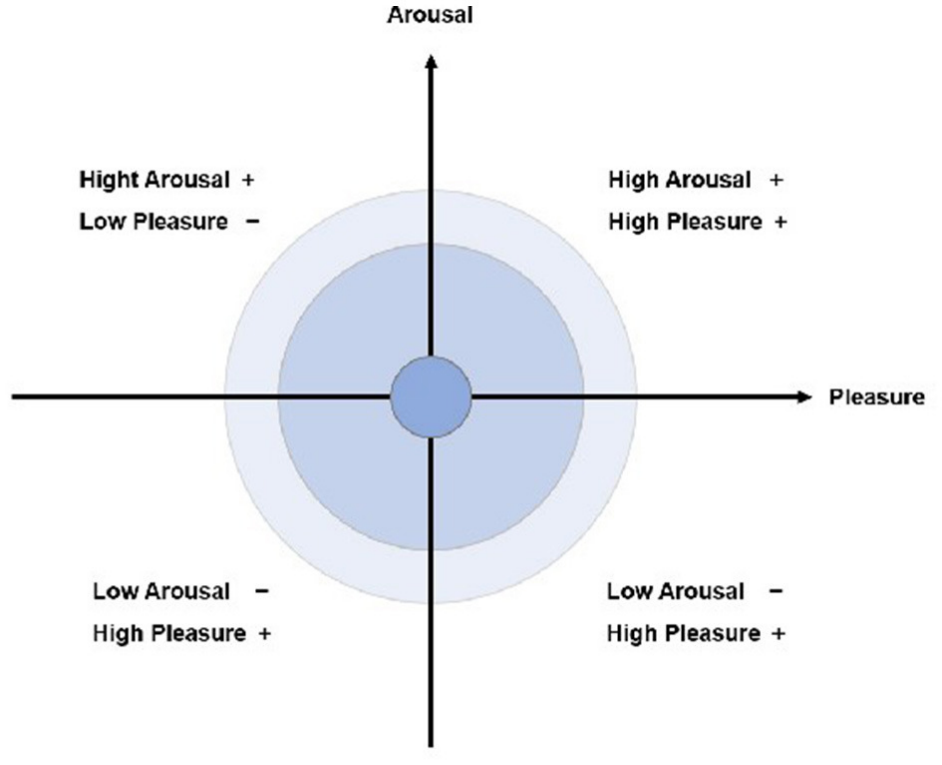
Framework based on emotional arousal model.

Physical and mental health is a topic of concern around the world (Fan et al., 2025), and many studies have shown that (Gillam, 2018; Slanzi et al., 2023) participation in arts activities is beneficial to physical and mental health. Arts on Prescription (AoP) promotes health and wellbeing through participatory creative activities (Bungay and Clift, 2010), with participants' emotional responses influenced by environmental, cultural (Pugh et al., 2022), and interpersonal factors. To analyze these dynamics, we adopt the Emotional Arousal Model (EAM) (Berlyne, 1960; Mehrabian and Russell, 1974), which emphasizes bottom-up sensory arousal (Sodhi et al., 2016) over top-down regulatory approaches (Figure 1). However, other emotional arousal models, such as Pleasure Arousal Dominance (PAD) (Bakker et al., 2014), are more commonly used in research related to a single sense, such as touch (Guendelman et al., 2024), human-computer interaction experience (Ke et al., 2025), and detecting human emotions (Schmitz-Hübsch and Becker, 2022) under intelligent interaction. EAM's ability to map arousal-pleasure states across creative stages (Russell, 1980) addresses limitations of static outcome measures in prior craft studies (Santini et al., 2023), aligning with the tactile-visual synergy of Portuguese Azulejo workshops. In previous studies, EAM has shown efficacy in depression treatment (Missirlian et al., 2005) and emotional enhancement in healthy populations (Jensen et al., 2023), supporting its use in “art prescriptions” for sub-optimal health (Sumner et al., 2019). Although the “art prescription” is not disease-specific, this study hopes to focus on the mental health of healthy people in conjunction with cultural specificities, and to prevent mental health problems through rich art therapies (Holt, 2020). Based on this framework, we expected that in this exploratory workshop, people's emotional arousal would start low (calm) in the initial touch stage and then rise to the final stage, with possible dips or stagnation in between.

Craft activities demonstrate strong associations with mental health and quality of life outcomes (Jensen and Bonde, 2018). Research (Santini et al., 2023) reveals a positive correlation between the frequency of art-driven craft participation and mental health indicators, with greater activity diversity linked to reduced depression risk. Given these findings, promoting craft activities in therapeutic settings has gained widespread endorsement (Horghagen et al., 2007). Previous research (Zhang et al., 2024) examining stress relief through ceramic workshops for college students provides valuable insights for the present investigation. While the therapeutic benefits of craft activities in promoting mental health are well-established (Bungay and Clift, 2010; Sumner et al., 2019), existing research predominantly emphasizes Western contexts and generic art forms such as painting or music. Although research on craft-based interventions acknowledges the importance of material engagement, with clay tasks demonstrating immediate calming effects on stress in university students (Van Lith et al., 2022), it often overlooks how culturally specific practices modulate emotional responses. Recent critiques highlight that AoP frameworks rarely integrate cross-cultural dynamics (Stepney, 2022), thereby limiting their applicability in multicultural settings such as Macau. Furthermore, while the Emotional Arousal Model (EAM) has been applied to explain sensory-driven behaviors in art therapy (Missirlian et al., 2005; Sodhi et al., 2016), its potential for dissecting stage-specific emotional trajectories within multisensory craft processes remains underexplored. This gap is particularly significant given emerging evidence that cultural immersion amplifies therapeutic outcomes through enhanced cognitive-emotional integration (Zhang et al., 2019; Daykin et al., 2020). Emotional arousal during craft creation is influenced (Silvia, 2005) by both the novelty of the creative medium and individual stress-coping capacity. During manual activities, participants' senses are stimulated to evoke various emotions and feelings, including patience, calmness, and surprise. Materials such as pigments and glazes used in Portuguese Azulejo tile painting provide rich tactile experiences, aligning with Xu et al.'s research demonstrating that artistic material preferences relate to psychological traits (Pöllänen, 2015), with individuals experiencing generalized anxiety showing a preference for soft (Xu and Huang, 2024), pliable media. However, research on cross-cultural workshop participation in art therapy remains limited. Macau provides a practical setting for Portuguese handicraft culture workshops, such as tile painting activities that emphasize multisensory engagement. Unlike traditional art forms like painting or music, these activities specifically engage both visual and tactile senses. The cultural specificity combined with sensory integration makes such workshops a distinct methodological approach to studying artistic and emotional expression. Research indicates that multisensory stimulation can enhance self-awareness (Pénzes et al., 2016), while variations in material fluidity, particularly through tactile interaction with mediums like ceramics or clay—have been observed to amplify emotional responses in individuals and groups (Nan et al., 2021) with indicators such as heart rate (Finck et al., 2023; D'Agostino et al., 2023) variability. This approach transcends mere technical skill acquisition, emerging as a profound mechanism for self-connection and emotional healing that addresses the current gap in cross-cultural art therapy research.

**Figure 2:**
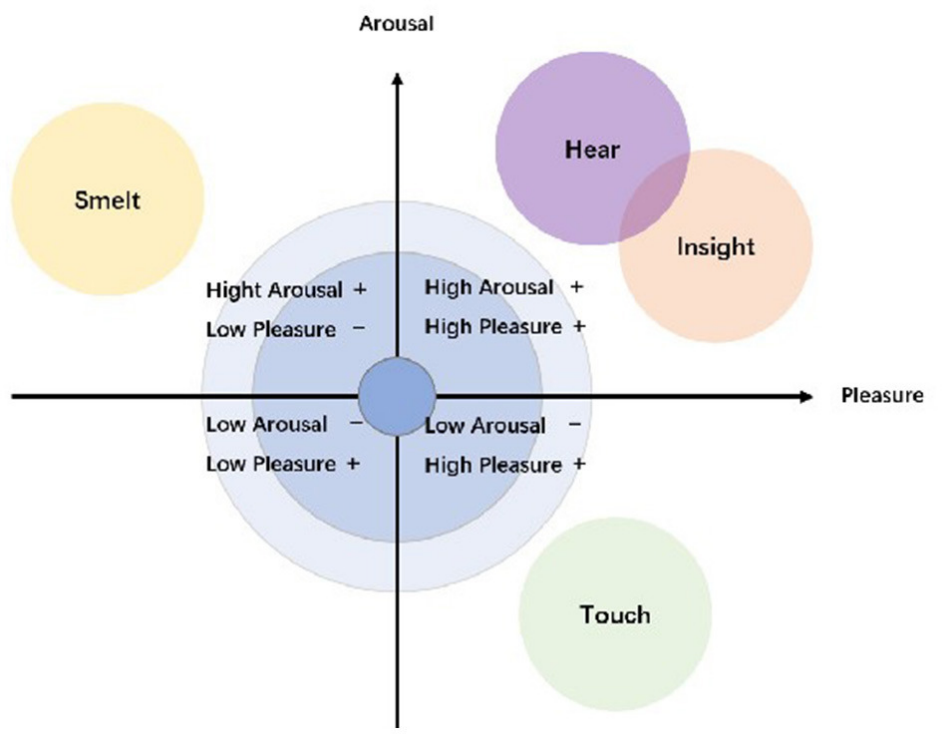
Categorization of different senses according to framework based on emotional arousal model.

Sensory processing across varied environmental contexts influences emotional responses distinctively, with valence contingent upon specific stimuli (Rodriguez and Kross, 2023). This underpins our hypothesis within the EAM framework, which categorizes emotional effects of sensory inputs in workshop settings (Figure 2). Interaction with diverse materials modulates both emotional states (Chen et al., 2021) and heart rate variability. The auditory and visual systems, directly connected to the amygdala, trigger rapid emotional arousal (Chen et al., 2021; Collignon et al., 2008) and frequently function synergistically in emotion recognition. In our study, participants responded to glaze colors, fluid materials, and tile-tapping sounds, categorizing these visual and auditory stimuli within the high-arousal, high-pleasure quadrant (Klein et al., 2020). Visual and auditory effects manifest almost immediately (Zhao and Rice, 2024), followed by tactile input (Cruciani et al., 2021), while olfactory stimuli exhibit greater complexity. The olfactory system's proximal connection to the hippocampus generates (Arshamian et al., 2020) more persistent emotional effects. According to Russell's circumplex model, olfaction—particularly regarding salient or hazardous odors (Russell, 1980)—falls into the high-arousal, low-pleasure quadrant. Affective touch, distinguished from general tactile input, evokes enhanced associative and emotional responses, reflecting its social and neural comfort functions (Beltrán et al., 2020). Given participants' unfamiliarity with Portuguese crafts, we propose that tactile stimuli in this context induce low-arousal, high-pleasure responses, suggesting novelty and relaxation.

Portuguese Azulejo painting, as a multisensory and cross-cultural artistic form, offers a unique avenue for emotional arousal and cultural cognition through its distinctive material tactility and traditional crafting processes. Specifically, the cultural cognition it engages pertains to the recognition and understanding of the historical and aesthetic context embedded in tile art. The repetitive manual actions and rhythmic motor patterns inherent in traditional craftsmanship have been shown to stimulate neural pathways associated with self-awareness (Cozolino, 2017; Nan, 2020). This study investigates the therapeutic potential of a culturally specific Azulejo tile painting workshop, grounded in the concept of “prescription art.” It seeks to elucidate how sensory engagement during various phases of creation contributes to the regulation of positive emotional states. Active participation in cross-cultural art forms, such as Azulejo, has been found to elicit more intense emotional responses than passive observation (Drake et al., 2023). While cultural background shapes individual interaction styles and feedback mechanisms within craft activities (Stepney, 2022), the underlying emotional regulation mechanisms appear to be consistent across cultural boundaries. Moreover, personal psychological identity is deeply influenced by cultural experience, which can in turn trigger physiological responses (Zhang et al., 2019; Beerse et al., 2019). Traditional culturally adaptive interventions often rely on cognitive behavioral therapy, emphasizing verbal exchange between therapist and client (Nagayama et al., 2019). In contrast, this study emphasizes non-verbal, hands-on workshops involving healthy adults in China, examining whether such culturally situated practices can promote positive emotional responses in a cross-cultural setting. To further investigate individual variability in emotional responses, this study considers the role of personality traits, particularly the Big Five dimensions and specific axes of the MBTI. Notably, the Thinking/Feeling and Introversion/Extraversion dimensions may significantly influence how individuals perceive and express emotions through artistic engagement. Engagement in cross-cultural art creation has been shown to enhance self-efficacy (Haiblum-Itskovitch et al., 2018), promote social connectivity (Daykin et al., 2020), and improve overall wellbeing. The process of craft creation also facilitates cultural integration (Kaimal et al., 2017a,b), and its emotional impacts have predictive value for the success of Art on Prescription (AoP) programs (Jensen et al., 2024). Similar to other forms of craft, Azulejo tile painting communicates deep emotional information through tactile perception and interaction with the surrounding environment (Shafir et al., 2020). This study applies the Emotional Arousal Mechanisms (EAM) framework to analyze how sensory experiences in Portuguese Azulejo workshops may lead to therapeutic outcomes. By focusing on tactile and multisensory cross-cultural engagement, it aims to identify mechanisms of emotion regulation, explore correlations between MBTI personality traits and color-emotion responses, and inform culturally informed art therapy practices in the multicultural context of Macau.

## 2 Methods

### 2.1 Participants

A total of 25 craft participants were recruited for the study, with 13 females and 12 males, from various locations throughout China, including Nanjing, Beijing, and Jiangsu (National Open Recruitment). The average age of the participants was 34.25 years (Table 1). The workshop was overseen by art specialists in Portugal, a senior researcher in the field of tile painting from Portugal, who provided expert guidance throughout the process. Furthermore, the materials and production processes utilized in the workshop were meticulously executed under the guidance and confirmation of professionals with backgrounds in fine arts and design.

**Table 1:** Participants' characteristics.

**Options**	**Frequency**	**Percentage (%)**
Female	13	52%
Male	12	48%
**Education**		
Mater	20	80%
PhD	5	20%
**Age**		
25–35	16	64%
36–45	8	32%
>45	1	4%
**Art experience**		
Inexperienced	5	20%
Slight knowledge	18	72%
Professional	2	8%

### 2.2 Outcomes measures

In this paper, the process of tile painting is divided into four stages involving four sensory experiences: visual, tactile, and a small amount of olfactory and auditory. We reviewed studies on art therapy and emotional arousal (Hadavi et al., 2022; Michalos and Kahlke, 2008), employing the subsequent three validated scales, each utilizing a five-point Likert scale, to assess participants' emotional shifts pre- and post-intervention. Subsequently, the results of the Wilcoxon signed-rank test were used to determine whether there was a difference in emotional changes before and after the workshop. Moreover, this study combines the Myers-Briggs Type Indicator (MBTI) personality (Myers et al., 1998) with the use of color and emotional expression in the works to deconstruct the role of personality traits on emotion-color associations in craft experience (Joshanloo, 2017), hoping to capture the subconscious emotional representation (Huang, 2024) in cross-cultural craft workshops. The MBTI analysis be used in this paper is still an exploratory attempt. Two researchers coded the units into the following points: first, the color type (such as blue or red). Second, the emotional keywords that participants were asked to answer in the questionnaire (for example: I just used a lot of blue because I felt calm inside). Finally, the two coders constructed the color-emotion association based on the relevant color psychology (Elliot and Maier, 2012) and literature.

### 2.3 Cultural cognitive and cultural preservation intent

The complete scale of cultural attachment has 20 questions (Hong, 2017), including questions about cultural identity, cultural preservation intent, cultural dependence, and cultural cognition. Since identity dependence requires long-term contact to accumulate and form (Hong et al., 2000), and cultural preservation intent can produce cognitive and emotional changes in a short period of time, we extracted questions about cultural preservation intent (Hong et al., 2016) to collect data. Participants need to select their degree of agreement with each statement on the spot, from 1 (“strongly disagree”) to 5 (“strongly agree”).

### 2.4 Subjective wellbeing scale (SWB)

Subjective wellbeing (SWB) constitutes a comprehensive construct encompassing individuals' affective states, satisfaction across various life domains, and overall assessments of life quality, which were quantified using five single-item indicators (Diener et al., 1985), each evaluated on a 1–5 Likert scale and analyzed independently. While primarily reflecting social wellbeing metrics, this measure also incorporates a subjective personal component that shapes an individual's perception (Arrondo et al., 2020) of their overall life experience.

### 2.5 The short-form positive and negative affect scale (PANAS)

The short-form Positive and Negative Affect Scale (PANAS) effectively measures emotional fluctuations (Thompson, 2007) and was used in this study. Negative affect (items 1–5) and positive affect (items 6–10) were rated on a 1–5 Likert scale. Research indicates that initial exposure to Portuguese handicrafts influences participants' positive emotions, shaping overall satisfaction and post-experience decisions (Zhao et al., 2020), supporting the role of positive affect in craft workshops.

### 2.6 Procedure

**Figure 3:**
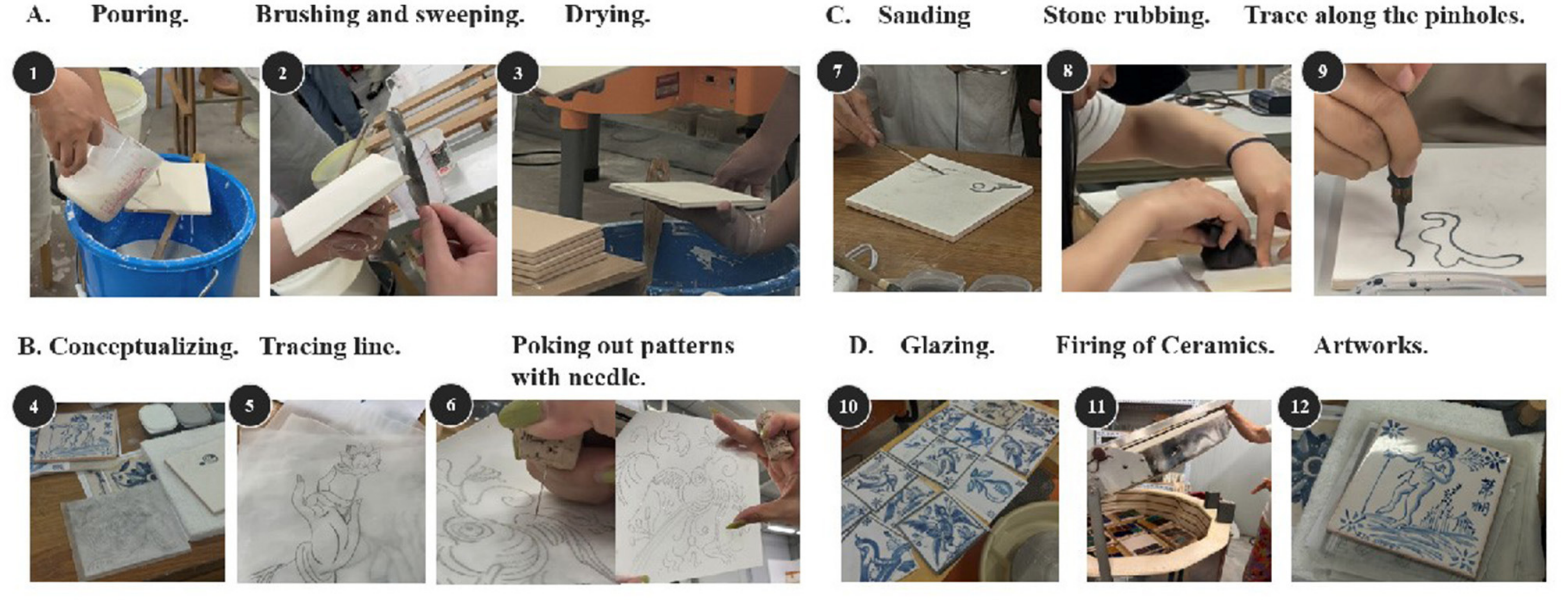
Different stages in the production of Portuguese tile paintings.

The workshop was conducted during the summer of 2024 at the ceramics studio in Macau over 2 months. The tile drawing production cycle comprises four distinct phases (Figure 3). The first phase involves brush sweeping, clay brushing, end flattening, and drying; the second phase encompasses motif conceptualization, line tracing, and pin-stamping; the third phase includes sanding, patting, and pinhole tracing; the final phase consists of celadon glaze application and firing. Participants were allowed to choose any colors and were instructed to select colors that expressed their momentary emotions. Notably, beyond designing tile patterns, specialists must produce blank tiles for subsequent drawing. Throughout all four phases, participation from both a Fine Arts professor and a Portuguese expert serving as lead lecturer is essential. It was considered a public involvement activity, and all participants gave informed consent before taking part.

### 2.7 Data analysis

In this study, all data were analyzed using IBM SPSS Statistics 29.0.2.0 software. Initially, descriptive statistics (mean, standard deviation) were calculated to illustrate participants' emotional fluctuations before and after the experience. The Kolmogorov-Smirnov and Shapiro-Wilk tests were conducted to assess the normality of the data distribution prior to further statistical analyses. Subsequently, the Wilcoxon signed-rank test was applied to assess the statistical significance of mood changes following participation (*p* < 0.05). Given the limited sample size, Pearson Correlation Analysis was employed to examine relationships between mood alterations and other relevant variables (culture cognition, wellbeing, culture preservation intent, emotion). To examine the relationship between production stage and emotional responses, Spearman's rank correlation coefficient analysis was conducted. In addition, all participants had already taken the MBTI official inventory and stated their four-letter type, and then, psychology and art therapy experts conducted a simple emotional analysis of the participants' works based on the Myers-Briggs Personality Profile (MBTI) during the creative process through the color selection of the works and emotional keywords in the interviews. We established the theme coding framework based on the actual data as follows: first, the color type (such as blue or red); second, the emotional keywords that participants were asked to answer in the questionnaire (such as “I just used a lot of blue because I felt calm”). Finally, we established a theme coding framework based on actual data including: color type (blue, red); emotional keywords from questionnaires (“I just used a lot of blue because I felt calm”); and color-emotion associations constructed by two coders using relevant color psychology literature (Elliot and Maier, 2012). Since only 12 of the collected works were allowed to be made public, the Art therapy experts analyzed participants' works using the Myers-Briggs Personality Profile (MBTI) and evaluated emotional changes through color selection and interview keywords.

## 3 Results

### 3.1 Primary outcomes

As evidenced in Table 2, pre-test data exhibited significant *p*-values (*p* < 0.05) in both the Kolmogorov-Smirnov and Shapiro-Wilk tests, indicating a non-normal distribution. Likewise, post-test data from the experimental group demonstrated significant *p*-values (*p* < 0.05) in normality tests (Table 3). Wilcoxon signed-rank tests were therefore conducted to assess the changes before and after the art-based intervention. Participants exhibited significant changes across several outcome dimensions following their participation in the Portuguese tile painting workshop. Specifically, cultural cognition scores increased significantly (W = 0, *p* < 0.001, r = 0.874), with the median rising from 15 [IQR = 11–16] to 17 [IQR = 16–20]. Similarly, wellbeing scores improved from a median of 37 [36–41] to 45 [40–51] (W = 0, *p* < 0.001, *r* = 0.874). A statistically significant increase was also observed in protective behavior (W = 0, *p* < 0.001, *r* = 0.874), with median scores increasing from 37 [35–39] to 43 [40–47]. Concerning affective outcomes, positive affect (PA) scores decreased slightly from a median of 39 [35–41] to 36 [34–38], though this change was statistically significant (W = 38, *p* = 0.004, *r* = 0.670). In contrast, negative affect (NA) did not reach statistical significance (*p* = 0.16), indicating that the workshop may have had limited impact on reducing negative emotions. Furthermore, regarding emotional variations within the subject group, an intriguing differential pattern emerged. PANAS positive affect scores increased significantly (*p* < 0.001), demonstrating the workshop's efficacy in promoting positive emotional experiences. However, PANAS negative affect did not reach statistical significance (*p* = 0.16), indicating that the art workshop may be more adept at enhancing positive emotions rather than directly diminishing negative ones. Descriptive statistics (means, standard deviations, medians, and IQRs) for all outcome variables are also provided in Table 3.

**Table 2:** The results of normality tests (K–S and S–W).

**Types**	**K–S**			**S–W**		
	**Group**	**Statistic**	***Df***	**Sig**	**Statistic**	**Sig**
Cognitive Changes	Pre	0.288	25	0.002	0.861	0.003
	Post	0.294	25	<0.001	0.813	<0.001
Wellbeing Institute	Pre	0.234	25	0.001	0.878	0.006
	Post	0.171	25	0.057	0.927	0.074
Preservation intent	Pre	0.138	25	0.200^*^	0.978	0.835
	Post	0.202	25	0.010	0.897	0.016
PANAS	Pre	0.103	25	0.200^*^	0.973	0.731
	Post	0.111	25	0.200^*^	0.964	0.497

Sig. values with an asterisk (^*^) indicate *p* > 0.05, suggesting normal distribution.

K–S, Kolmogorov–Smirnov; S–W, Shapiro–Wilk; Sig., Significance (p-value).

**Table 3:** Wilcoxon signed-rank test between pre- and post-workshop experience and different influencing mood elements.

**Types**	**W Statistic**	***p*-value**	**Effect size *(r)***	**Mean (SD)**	**Mean (SD)**	**Median [IQR]**	**Median [IQR]**
				**Pre**	**Post**	**Pre**	**Post**
Cognitive changes	0	<0.001^***^	0.874	13.64 (3.13)	17.68 (2.59)	15.0 [11.0–16.0]	17.0 [16.0–20.0]
Wellbeing institute	0	0.003^**^	0.874	37.64 (5.42)	45.28 (6.71)	37.0 [36.0–41.0]	45.0 [40.0–51.0]
Preservation intent	0	<0.001^***^	0.874	37.08 (5.06)	44.00 (6.19)	37.0 [35.0–39.0]	43.0 [40.0–47.0]
PANAS-NA	0	0.16	0.874	28.04 (4.25)	27.52 (3.92)	27.0 [26.0–31.0]	27.0 [26.0–31.0]
PANAS-PA	38	<0.001^***^	0.67	37.84 (4.84)	35.64 (4.66)	39.0 [35.0–41.0]	36.0 [34.0–38.0]
PANAS	34	0.003^**^	0.692	65.88 (8.87)	63.16 (8.08)	68.0 [61.0–72.0]	64.0 [60.0–68.0]

Correlation is significant at the p < 0.05 level. ^**^Correlation is significant at the p < 0.01 level. ^***^Correlation is significant at the p < 0.001 level.

### 3.2 Secondary outcomes

To examine the relationship between the different stages of tile production and emotional responses, Spearman rank correlation coefficient analyses were conducted, as shown in Table 4. Several notable findings emerged across the four stages of Azulejo craft production, revealing strong negative correlations between production stage and negative emotional states, particularly for *Nervous* (ρ = −0.850) and *Caution* (ρ = −0.850), suggesting that these emotions consistently declined as participants progressed through the workshop. In contrast, positive emotions were weakly and positively correlated with production stage, with *Excited* showing a moderate positive correlation (ρ = 0.400), while *Happy* and *Relaxed* showed weak positive correlations (ρ = 0.200). Although only the correlations between *Excited* and both *Joyful* and *Nervous* reached statistical significance (*p* < 0.05), this may be due to the small number of stages analyzed. However, the size of the correlation coefficients suggests meaningful emotional patterns. These patterns are worth considering in the context of craft therapy, which relies on deep emotional engagement. Notably, the variance in emotional responses was strongly positively correlated with progression across stages (ρ = 0.800), indicating that emotional variability increased as participants approached the completion of their work.

**Table 4:** Spearman rank correlation coefficient analysis results of the relationship between different production stages and emotions.

**Emotion type**	**Spearman's ρ**	***p*-value**	**Mean-whole progress**
Excited	0.40	0.04^*^	3.83
Joyful	0.20	0.02^*^	3.91
Relax	0.20	>0.05	3.86
Nervous	−0.85	0.03^*^	2.64
Pressure	0.40	>0.05	2.43
Caution	−0.85	>0.05	3.24
Mean	0.40	>0.05	/
Variance	0.80	>0.05	/

^*^Correlation is significant at the *p* < 0.05 level. ^**^Correlation is significant at the *p* < 0.01 level. ^***^Correlation is significant at the *p* < 0.001 level.

**Figure 4:**
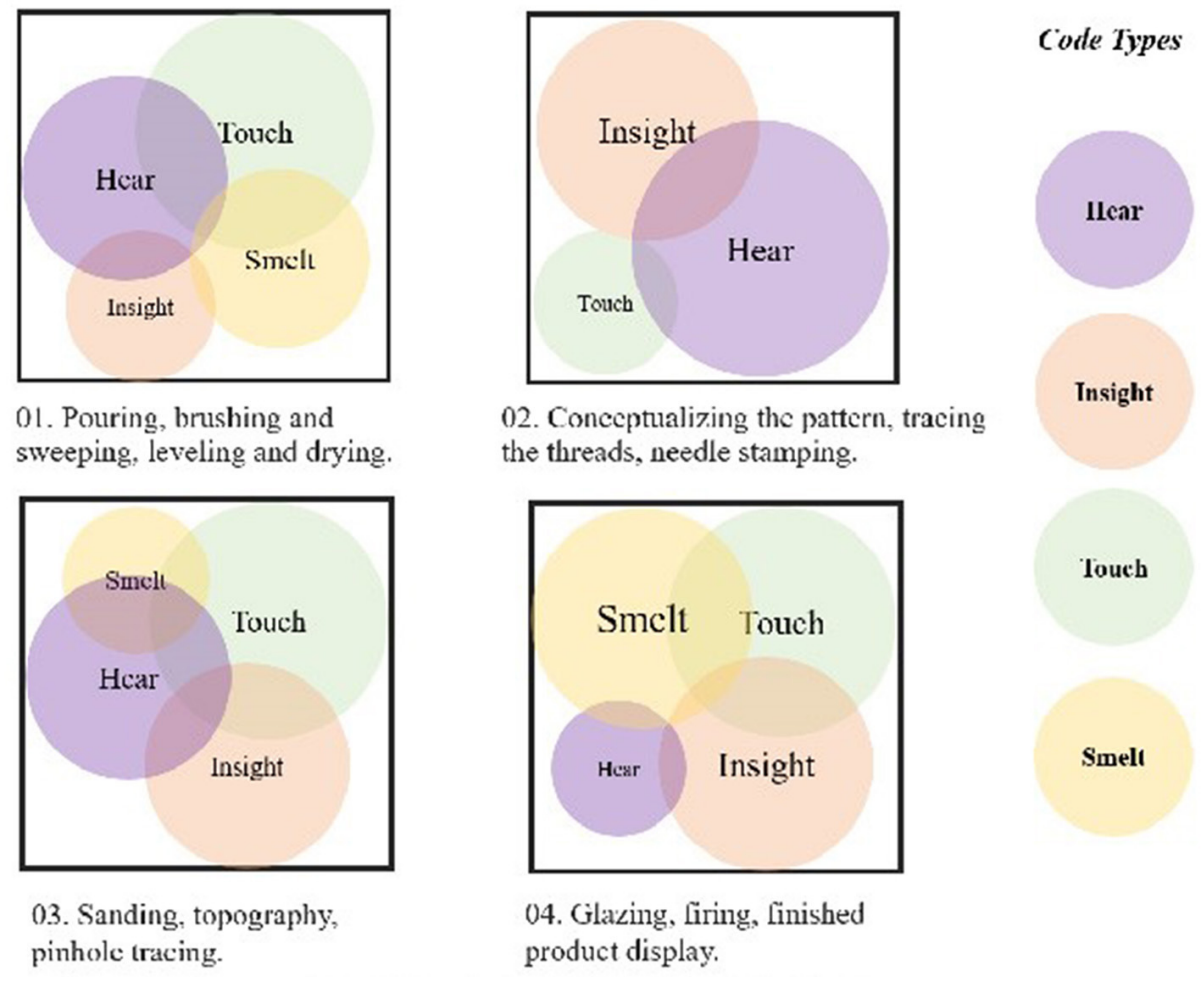
Synergy of sight, touch, hearing and smell in crafts.

Moreover, as shown in Table 5, notable emotional fluctuations were observed across the four production stages. The average scores for positive emotions (including excitement, joy, and relaxation) followed a biphasic trajectory throughout the process (initial calming followed by end-heightened arousal), whereby We attempted to show that the visual, tactile, auditory, and olfactory senses involved in the participants' emotions were co-expressed and that there were differences in sensory dominance at different stages (Figure 4). Participants initially reported moderately high levels of positive affect during the early stages of engagement (casting, scrubbing, and drying; *M* = 2.79). These levels slightly decreased during the conceptualization and outlining phase (*M* = 2.57), reached their lowest during the intermediate restoration and adjustment phase (*M* = 2.50), and then rose sharply during the final stage of glazing, firing, and finishing (*M* = 3.25). In contrast, negative emotions (such as tension, stress, and a sense of burden) demonstrated a general downward trend across the first three stages (declining from *M* = 1.91 to *M* = 1.68), but showed a slight increase in the final phase (*M* = 1.71). Participants' tension tended to rise in the final stage of the process, potentially reflecting the demands associated with task completion.

**Table 5:** Average positive and negative emotional responses across production stages.

**Stage**	**Description**	**Positive emotion Avg**	**Negative emotion Avg**
ONE	Pouring. Brushing. Drying	2.79	1.91
TWO	Conceptualize. Tracing. Poking	2.57	1.74
THREE	Sanding. Rubbing. Tracing	2.50	1.68
FOUR	Glazing. Firing. Finished	3.25	1.71

Positive emotions include Excited, Joyful, and Relax; Negative emotions include Nervous, Pressure, and Caution (From PANAS Scale).

**Figure 5:**
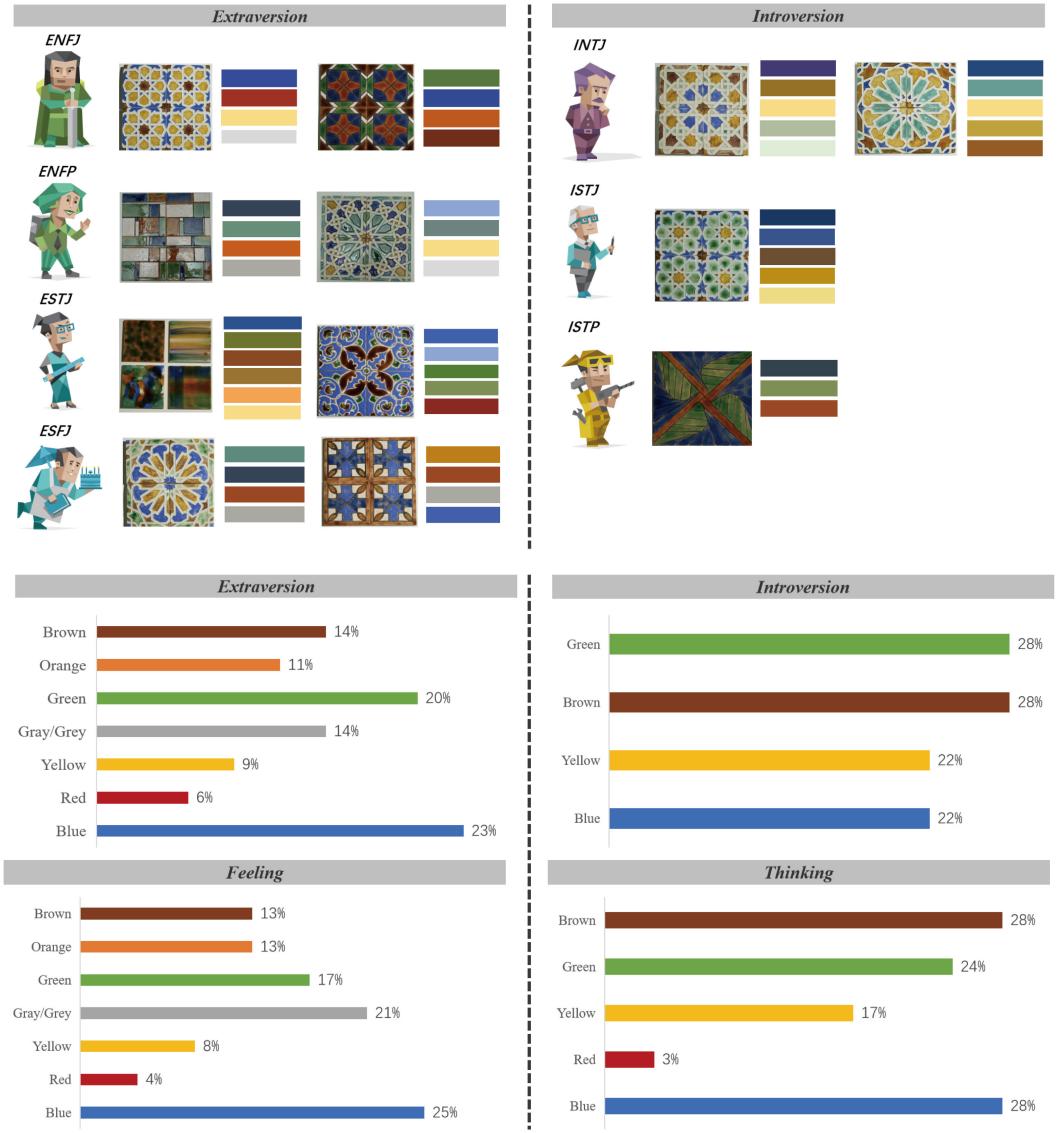
Based on the picture and emotions under MBTI & Color tendencies under the influence of MBTI traits.

The MBTI does not directly determine color mood, but it does provide a cognitive-emotional perspective that explains different color choices. We sorted out the emotional keywords of the participants during the coloring stage, and based on Elliot's research results on the correspondence between colors and emotions (Elliot and Maier, 2012), we constructed the color-color correspondence shown in Table 6. All the content revolved around their subjective understanding of colors and emotions during the painting process. The color tendencies and emotional associations of different personality traits are shown in Table 7. This time, two independent researchers conducted the coding, and the consistency was evaluated using Cohen's Kappa, with a result of κ = 0.76, indicating good coding consistency. Analysis of participants' color choices revealed systematic patterns between MBTI personality traits and emotional expression. Feeling-type (F) predominantly selected blue combinations, with Figure 5 confirming their strong preference for blue (25%), green/grey (21%), and brown (13%). These selections corresponded with emotional associations of “calm,” “patience,” and “security.” Thinking-type (T) exhibited a marked preference for brown (32%) and blue (25%), with yellow (14%) also featuring prominently. Extraverted-types (E) tended toward energetic color combinations, with blue (23%) and green (20%) dominating their palette. Introverted participants (I) selected colors associated with patience and stability, distributing their preferences equally among blue, yellow, and brown (each 25%), while notably avoiding red completely. These consistent alignments between MBTI characteristics and color-emotion associations suggest personality traits may influence both color selection and emotional processing.

**Table 6:** The relationship between glaze color and craftsman's emotional association based on the results of color psychology.

**Color**	**Common emotional associations**
Red	Passion, Excited, Energy
Blue	Calm, Melancholy, Patience
Yellow	Happy, Hope, Persistence
Green	Relaxation, Balance, Naturalness
Gray	Calmness, dullness, Reserve
Brown	Stable, Security, Introspection

**Table 7:** Correspondence between glaze colors and emotional association ns in the workshop.

**No**.	**MBTI**	**Main color**	**Color-emotional semantics**	**Emotion**	**Color proposition classification**
P1	ENFJ	Blue. Yellow	Calm-Passionate	Positive	E-F
P2	ENFP	Green. Gray	Relax-Dullness	Positive/Neutral	E-F
P3	ESFJ	Blue. Brown	Calm-Security	Positive	E-F
P4	INTJ	Yellow. Brown	Hopeful-Melancholy	Positive	I-T
P5	ISTP	Blue. Red	Patience-Energy	Positive	I-T
P6	ESTJ	Brown. Blue	Stable-Melancholy	Positive	E-T
P7	ESFP	Blue. Red	Calm-Energy	Positive	E-F
P8	ISTJ	Blue. Yellow	Patience-Persistence	Positive	I-T
P9	INFP	Green. Brown	Relax-Security	Positive	I-F
P10	ESTP	Red. Blue	Energy-Melancholy	Positive	E-T
P11	ISFJ	Blue. Yellow	Patience-Persistence	Positive	I-F
P12	INTP	Blue. Green	Calm-Relax	Positive	I-T

E, Extraversion; F, Feeling; I, Introverted; T, Thinking.

## 4 Discussion

This study employs emotion-tracking methodologies to evaluate the artistic experience of participants in cross-cultural workshops, assessing its correlation with health regulation. This facilitates real-time health feedback from art workshop engagement, potentially serving as a mechanism for future health modifications in targeted applications.

### 4.1 The healing potential arising from multisensory experiences

In the process of creating Portuguese Azulejo tiles, the coordination of clay, pigments, and tools requires continuous limb coordination to shape raw tiles before drawing, with these soft and flexible art materials—characterized by lightness and expressiveness—offering timely benefits for participants experiencing tension (Nan et al., 2021) and anxiety. Compared to singular visual arts, the synchronous effect of sensory information amplifies emotional arousal and enhances (Aron et al., 2012) sensory processing sensitivity. Additionally, the flowing texture of brushing and mixing clay stimulates a free and comfortable sensation; this process (Sweeney and Homeyer, 1999) even adjusts the sympathetic and parasympathetic nervous systems. In fact, Man et al. (2014) found that long-term engagement in art workshops improves hedonic capacity, vivid inner imagery, and flow states. The process of mixing pigments (Holt, 2018), brushing clay, and imprinting toner generates a rich continuum of sensations, where hand contact with materials elicits positive or negative feelings based on varying pressure. Tactile perception through the skin forms mental imagery, as if awakening “eyes” in the skin, broadening the scope of emotional arousal (Grunwald, 2008). The observed emotional fluctuations align with the sensory-mediated framework proposed in our theoretical model, wherein the early touch-dominant stages and the final vision-dominant stages of production elicit the most pronounced positive emotional responses. The intermediate phases of production, which demand greater precision and technical focus, were associated with comparatively subdued emotional states, possibly reflecting the heightened cognitive and attentional demands characteristic of these stages. Notably, despite the overall positive tone in the final stage, participants' tension tended to increase. This parallels findings from the performance psychology literature, such as the growth in anxiety observed between anticipation and task completion in speech performance tasks, characterized by heightened arousal (Harris et al., 2023) when finishing goal-oriented actions. This increase may be attributed to heightened anticipation and the pressure to finalize the product, consistent with prior findings on performance-related stress in evaluative or outcome-oriented contexts. Overall, these findings support the hypothesis that emotional arousal systematically varies across the sensory-dominant stages of the Azulejo production process, with emotional elevation particularly evident during the initial material engagement and the final artistic culmination. Here, touch encompasses the abstract sensation of bodily experience (Elbrecht and Antcliff, 2014), and these materials are intertwined with multidimensional sensory factors that collectively influence the impact of art on the human psyche and quality of life. This relates to the conjecture we attempted in the Introduction that the “tactile” perception of art materials is related to the enhancement of certain positive emotions (pleasure).

### 4.2 The appeal of cross-cultural craft activities

Previously, teams addressing psychological issues during the COVID-19 period used multimodal art creation across nations to alleviate distress, finding that (Elbrecht and Deuser, 2012) art experiences effectively redirect negative attention. Although emotion research focuses on facial and vocal expressions, extending exploration of multicultural dimensions through bodily senses yields distinct outcomes (Porto et al., 2021), with physical movements and postures comprehensively promoting (Corem et al., 2015) emotional regulation. In most group-based craft therapy workshops (Leichner et al., 2014), participants are encouraged to work together as a group to improve social skills (Dael et al., 2012) and reduce loneliness. In addition, we fully agree that practical applications still need to adjust the form of craft participation in combination with local cultural characteristics. Gender and age may indirectly affect emotional responses through variables such as personality traits and technology familiarity (Bandura, 1997), but existing data have not found that they significantly interfere with the core mechanism. In fact, cultural adaptation of behavioral and health interventions is challenging, and health interventions around the world (Fischer et al., 2024) are working on this.

Moreover, the creation of Azulejo tiles demonstrates how thoughtful use of color and material can effectively convey emotion (Cavanagh et al., 2020). Warm hues are particularly associated with feelings of vitality, enthusiasm, and optimism (Çetinkaya et al., 2019). Research supports a clear link between color and emotion: artists often express their inner emotions through their work (Hinz, 2009); specific emotional responses can be tied to particular colors (Kaya and Epps, 2004); and cultural meanings further shape emotional interpretations of color—such as yellow with hope, black with despair (Meier and Robinson, 2005), and green with relaxation. These findings explain workshop observations where participants preferred bright colors while displaying positive emotions, suggesting color choices both express and reinforce emotional states (Wilms and Oberfeld, 2018). Artistic aesthetics feature bottom-up stimulus traits where artwork elements directly trigger intuitive sensory responses (Valdez and Mehrabian, 1994), their cognitive-level similarity offering clues for exploring intrinsic art perception mechanisms (Pelowski et al., 2017). These findings align with Fancourt's work demonstrating that art participation effectively enhances self-identity, elevates self-esteem, and cultivates agency (Fancourt et al., 2019), explaining the predominance of positive emotions observed during the workshop. This craft interaction, characterized by immediate feedback, increases happiness and pleasure emotions (Hermann and Geneseo, 2023) by bolstering participants' self-esteem. Fancourt's team emphasized that “art-making activities inherently promote emotion regulation strategies through embodied practice.” However, in this cross-cultural Portuguese Azulejo workshop, observational data showed transient negative emotions during technically demanding stages (such as “brushing” techniques and “stone friction” procedures). This aligns with skill acquisition theory, which posits that initial performance failures may temporarily impede emotion regulation (Bandura, 1997), explaining the persistent unmitigated negative emotions across different stages.

Despite technical challenges, the workshop's positive emotional outcomes validate Jiang's findings on the unique emotional (Jiang et al., 2025) benefits of handicrafts. This artistic form transcends cultural boundaries, suggesting craft-making functions beyond specific technical or cultural barriers through broader creative mastery and aesthetic engagement. We agree that practical applications should adapt craft participation to local cultural characteristics. Gender and age may indirectly affect emotional responses through variables like personality traits and technology familiarity (Bandura, 1997), but existing data show no significant interference with the core mechanism. Cultural adaptation of behavioral and health interventions remains challenging (Fischer et al., 2024), with global health interventions actively addressing this issue.

### 4.3 Emotional differences triggered by MBTI personality traits

This study investigates the emotional effects of multisensory engagement in Portuguese Azulejo craft workshops and examines correlations between MBTI personality traits, color selection, and emotional expression. Colors convey both cultural significance and universal psychological implications (Fetterman et al., 2015). Empirical research (Rusting and Larsen, 1998) supports the application of cognitive-affective models in color selection processes. Notably, Xue's machine learning analysis identified significant correlations between personality traits and color preferences (Xue and Ding, 2025), consistent with Ou et al.'s findings that the extraversion-introversion dimension substantially influences color perception (Ou et al., 2004). Specifically, extraverted individuals demonstrate preferences for highly saturated hues, while introverted individuals gravitate toward cooler, less saturated tones.

Although the MBTI is not designed to directly measure emotions, it offers an interesting perspective, particularly in the multisensory environment created in this workshop. In craft creation, these traits (Geen, 1984) influence emotional regulation by enhancing emotional expression. Participants' color choices, driven by their MBTI profiles, reveal various emotional associations. For example, individuals with a Feeling (F) preference tend to favor colors such as blue, green, and brown, which are associated with emotions of calmness, security, and patience, reflecting the warm and soothing qualities these colors evoke in an emotional context. On the other hand, Thinking (T) types, who tend to process information analytically, exhibit a preference for colors like brown, yellow, and blue, which are linked to neutral emotions such as calmness and stability, aligning with Lee's findings (Lee and Lee, 2021) that emotional stability is positively correlated with cooler tones. The preference for yellow, associated with hope and persistence, may suggest optimism and rational problem-solving behaviors in this experience. Extraverted (E) participants tend to choose colors, which our findings collectively support a link between cultural cognition, emotion, and behavioral intentions in art interventions to nature and are associated with vitality, closeness (Afzali et al., 2020; Jue and Ha, 2022), and social engagement. In contrast, Introverted (I) participants prefer softer hues like green and yellow, indicating a tendency toward calmer or more enduring emotional states (Lee and Lee, 2021) and heightened sensitivity (Christensen et al., 2020) to material textures. These patterns indirectly support the idea that extraverts are more likely to be drawn to high-energy colors, while introverts tend to favor cooler, less stimulating hues. The workshop environment, characterized by multisensory stimuli (visual, tactile, and auditory), significantly influenced emotional arousal, as indicated by participants' color preferences. Tactile interaction with materials, alongside visual cues, elicited varied emotional responses (e.g., curiosity, joy, frustration). Extraverted individuals, known for their responsiveness to external stimuli, may demonstrate heightened excitement and joy during dynamic phases of tile painting, reflected in their preference for highly saturated colors. Furthermore, extraverts are likely to experience greater positive emotional arousal during interactive activities. Conversely, introverted individuals may perceive the same activity as more soothing, engaging in introspective emotional regulation during quieter moments.

These findings suggest that personality traits may influence how individuals emotionally interact with art materials, especially in cross-cultural, sensory-rich environments. The initial exploration of MBTI and color emotions provides an interesting perspective for emotional healing interventions based on the premise of art and crafts, especially in short-term, low-threshold treatment programs like “prescription art.”

## 5 Strengths and limitations

This study demonstrates that Arts on Prescription (AoP) effectively improves psychosocial wellbeing through quantitative measures. However, we examined only short-term effects and lack longitudinal follow-up data on sustained emotional arousal impacts, despite verifying immediate positive emotional effects. We attempted 30-day post-intervention follow-ups but encountered significant limitations. Most healthy participants reported emotional responses as immediate workshop experiences and declined to provide follow-up feedback. Future research will address these limitations by combining physiological indicators (heart rate variability, skin conductance) with psychological assessments for objective emotional arousal verification. Studies will expand sample diversity across cultural backgrounds and age groups to examine demographic moderating effects (Adams and Osgood, 1973) and enhance cross-cultural art therapy frameworks. Including control groups will strengthen evidence for AoP's long-term wellbeing effects and underlying mechanisms.

## 6 Implications for practice and further research

This study confirms the therapeutic value of cross-cultural craft participation in regulating emotions through multisensory integration. It significantly enhances cultural identity and subjective wellbeing, while effectively stabilizing emotional fluctuations. It also offers a theoretical foundation for culturally informed art therapy that considers individual differences, specifically, the empirical connection between MBTI personality types and color-emotion associations. Practically, these insights enable art therapists to tailor material choices according to MBTI profiles (e.g., using tactile materials for introverts or bright colors for extroverts), refine emotional interventions by aligning sensory experiences with emotional stages (e.g., calming tactile phases followed by energizing visual ones), and support cultural institutions in designing therapeutic experiences that combine craftsmanship with emotional healing. Although centered on the case of Azulejo, the study's multidimensional and stage-based approach offers broadly transferable insights. It can inform art-based interventions across diverse settings, including hospitals (Yang et al., 2023), elder care facilities, educational institutions, community centers (Mosadeghrad et al., 2022), and corporate wellness programs. By integrating cultural immersion, sensory engagement, and personality-based customization, this research increases the actionable nature of the Art of Prescription and encourages interdisciplinary collaboration (Michelle et al., 2022) to expand its global community impact.

## 7 Conclusion

The multisensory engagement framework demonstrated stage-specific emotional patterns, with tactile-dominant initial phases and visual-dominant completion phases eliciting the most robust positive responses. While cultural art participation facilitates emotional expression, catharsis, and enhanced verbal communication, a systematic deconstruction of the neurobehavioral mechanisms of multisensory art experiences and quantification of perceptual channel effectiveness in emotional regulation are warranted. Furthermore, MBTI personality traits correlated with distinct color-emotion associations. These findings offer insights for developing targeted therapeutic craft interventions, providing promising directions for cross-cultural health promotion programs.

## Data Availability

The original contributions presented in the study are included in the article/supplementary material, further inquiries can be directed to the corresponding author.
